# Memory function in autoimmune encephalitis: a cross-sectional prospective study utilising multiple memory paradigms

**DOI:** 10.1007/s00415-024-12520-z

**Published:** 2024-06-25

**Authors:** Sarah P. Griffith, Robb Wesselingh, Nabil Seery, Tiffany Rushen, Chris Kyndt, Brian Long, Udaya Seneviratne, Tomas Kalincik, Katherine Buzzard, Helmut Butzkueven, Terence J. O’Brien, Rubina Alpitsis, Charles B. Malpas, Mastura Monif, Australian Autoimmune Encephalitis Consortium

**Affiliations:** 1 Department of Neurosciences, Central Clinical School, Faculty of Medicine, Nursing and Health Sciences, Monash University, Level 6, Alfred Centre, 99 Commercial Road, Melbourne, VIC 3004 Australia; 2 Department of Neurology, Alfred Health, Level 6, Alfred Centre, 99 Commercial Road, Melbourne, VIC 3004 Australia; 3 Department of Neurology, Melbourne Health, 300 Grattan Street, Parkville, VIC 3050 Australia; 4 Department of Neurosciences, Eastern Health Clinical School, Monash University, Box Hill Hospital, Melbourne, VIC Australia; 5 Neuropsychology Unit, Monash Health, 246 Clayton Road, Clayton, VIC 3168 Australia; 6 Department of Neurosciences, Monash Health, Clayton Road, Clayton, VIC 3168 Australia; 7 CoRE, Royal Melbourne Hospital, The University of Melbourne, Victoria, Australia; 8 Melbourne School of Psychological Sciences, The University of Melbourne, Victoria, Australia

**Keywords:** Autoimmune encephalitis, Memory, Neuropsychology, Cognition, Neuroinflammatory

## Abstract

**Background and objective:**

Autoimmune encephalitis (AE) is often associated with clinically significant memory impairment. This study aimed to evaluate memory in a cross-sectional prospective AE cohort using multiple memory paradigms.

**Methods:**

52 patients (50% seropositive) meeting Graus criteria for possible AE were prospectively recruited between October 2019 and August 202. A comprehensive examination of memory was performed, including tests of supraspan verbal memory (list learning), logicosemantic memory (story learning), figural memory (learning of geometric designs), and verbal associative learning (verbal paired associates). Memory scores were compared to demographically adjusted normative data. Pattern analysis was conducted to assist in the identification of patterns in memory performances.

**Results:**

Mean memory scores were not significantly below the normative mean. At an individual patient level, over 20% of the cohort exhibited impaired delayed figural memory, supraspan verbal memory learning and recall. Observed performances were significantly below expected performance for story learning (*p* = 0.017) and recall (*p* = 0.003), figural recall (*p* < 0.0001), initial acquisition (*p* < 0.001) and final acquisition of a list (*p* < 0.001) and all delayed recall measures of the list (*p* < 0.00001). 54.76% of patients exhibited intact psychometrics, and 16 distinct patterns of impairment emerged, indicating variability in memory outcomes.

**Discussion:**

While statistical evidence for memory impairment did not emerge at an aggregate level, a proportion of patients present with evidence of abnormal memory performance on psychometrics. Variability in impaired memory measures argues for an individualised patient-focused approach to clinical assessment in AE. Future research should validate these findings with a larger sample size and explore the relationships between memory profiles and other cognitive functions.

**Supplementary Information:**

The online version contains supplementary material available at 10.1007/s00415-024-12520-z.

## Introduction

Autoimmune encephalitis (AE) comprises a group of neuroinflammatory disorders affecting the central nervous system. Patients present acutely with a broad spectrum of symptoms, including cognitive changes, seizures, motor disturbances, and/or psychiatric features. Several neuronal cell-surface target antigens have been associated with developing AE, including *N*-methyl-d-aspartate receptor (NMDAR), leucine-rich glioma-inactivated 1 (LGI-1), contactin-associated protein-like 2 (CASPR2), IgLON5, γ-Aminobutyric acid A/B (GABA A/B) and dipeptidyl-peptidase-like protein-6 (DPPX). These are often referred to as ‘seropositive’ AE [1]. The term ‘seronegative’ AE describes patients who meet the criteria for possible AE but have no identifiable antibody.

Previous studies have reported variable memory outcomes across different ab-mediated AE subtypes, with reports of both normal and abnormal memory function [2–7]. The heterogeneous findings might be due to the different approaches used to detect memory impairment. First, different comparator data have been used, including matched healthy controls and normative datasets. Second, reported metrics of memory function have varied, with some studies reporting learning scores while others reporting delayed recall scores. Third, differing definitions of impairment have also been employed, including liberal criteria such as one standard deviation below the normative mean. Finally, different memory paradigms, such as logicosemantic material (e.g. narrative prose) or list-learning tests, have been utilised separately but not often in the same cohort. Although a systematic comparison of memory tests in neuroimmunology populations has not been performed, an exploratory study in epilepsy patients suggested that a test of logico-semantic memory, list-learning with embedded semantic categories, and list-learning without categories could not be considered interchangeable [8]. This was owing to the varying demands on non-memory functions, including differences in semantic processing and memory organisation [8]. Moreover, the complexity of interpreting memory deficits is compounded by the potential for different outcomes across various cognitive tests, as highlighted in the above study [8]. Such nuances underscore the risk of misinterpreting impairment or change in a test, especially if it inadvertently captures more than one cognitive domain. This emphasises the importance of comprehensive assessment approaches in understanding memory deficits, particularly in the context of neuroimmunology populations like AE, where memory deficits may coexist with impairments in attention and executive functions, contributing to functional memory difficulties. This is particularly important as there is evidence of a number of cognitive deficits in AE patients [4, 5, 7, 9, 10]. Collectively, these factors limit the ability to draw clear conclusions regarding memory impairments after AE.

Understanding the specific type of memory outcomes is crucial for guiding targeted clinical assessment and directing future research, including investigating correlations with imaging and serological and cerebrospinal fluid biomarkers. For example, work in dementias has demonstrated the importance of deriving cognitive patterns to differentiate between distinct neurological diseases, as relying solely on clinical criteria such as ‘memory impairment’ may lack diagnostic precision [11]. This is best demonstrated in differences in memory characterisation in patients with Alzheimer’s disease and Lewy Body Dementia (LBD), where the episodic memory impairment in AD is characterised by a diminished ability to encode new material, affecting both recall and recognition of material [12]. In contrast, the nature of memory impairments in LBD is characterised as one of retrieval rather than encoding difficulties [12]. Considering the importance of distinguishing cognitive patterns in various neurological diseases due to their impact on accurate diagnosis, individualised treatment strategies, prognosis, and potential contributions to research and drug development, understanding memory profiles is imperative.

In this study, we conducted comprehensive memory examinations on patients at least 6 months post-diagnosed AE, utilising a battery of memory tests across various paradigms. The objective was to investigate the profile of memory deficits following AE, examine the frequency of memory test deficits, and identify patterns of memory outcomes.

## Methods

### Participants

Patients were identified and recruited through outpatient neurology clinics at four major metropolitan health services in Melbourne, Australia—Alfred Health, Monash Health, Eastern Health, and Melbourne Health as part of the larger Australian Autoimmune Encephalitis Consortium (AAEC) between October 2019 and August 2022. Inclusion in the database is described elsewhere [13–15]. Seropositive patients had antibody testing conducted as per their corresponding hospital's procedures at the time of their initial presentation. For AE associated with specific antibodies, those antibodies had to be present in the CSF and/or serum with the highest sensitivity. In this study, those with onconeural antibodies were not included.

Participation in the neuropsychological arm required patients to meet the criteria for possible AE as per the criteria of Graus and colleagues [1] at least 6 months after the diagnosis of AE. The initial presentation of AE could have occurred at any time in the past, as long as the participant was over the age of 18 at onset and had English as their primary language. Those with a current diagnosis of a neurodegenerative disease or currently under investigation for a possible neurodegenerative disease (e.g., Alzheimer’s disease) were excluded. Patients with other significant neurological comorbidities that could be contributing to cognitive profiles, including tumors and strokes, were excluded, however patients with epilepsy arising from the disease were included. None of the patients had a history of developmental language disorder or were diagnosed with an intellectual disability. Patients or their person(s) responsible (in cases where the patient could not consent themselves) provided informed consent.

### Standard protocol approvals, registrations, and patient consents

The central Human Research Ethics Committee at Alfred Health approved the study (HREC/17/Alfred/168).

### Procedure

All patients underwent neuropsychology assessment, including a semi-structured clinical interview conducted by a clinical neuropsychologist (S.G.). Sociodemographic variables (age, gender, and years of education) and clinical information were collected during the interview. Other clinical and paraclinical data was obtained through the AAEC database, most of which is derived from data from the initial hospital admission. Note that not all patients have data for all measures. Data regarding immunotherapy treatment was collected, where the first line was classified as IVIg and/or corticosteroids; the second line included rituximab or cyclophosphamide; and the third line included tocilizumab or bortezomib. Data on other clinical and paraclinical variables were collected from medical records when available. They included ICU admission (y/n), mRS (modified Rankin Scale [16]) at discharge and the number of antiseizure medications (ASM) at the time of assessment. Due to local COVID-19 restrictions during part of the recruitment phase, five patients were assessed via telehealth. Other reasons for incomplete assessments included physical limitations (*n* = 2), too cognitively impaired for aspects of the battery (*n* = 4), telehealth difficulties (*n* = 2), and time limitations (*n* = 2).

### Materials

The following memory tests were administered to patients and were embedded in a larger cognitive battery—California Verbal Learning Test—2nd Edition (Standard Form) [17], Wechsler Memory Scale—4th Edition [18] (Logical Memory and Visual Reproduction subtests) and Wechsler Memory Scale—1st Edition [19] (Verbal Paired Associates Subtest). Further details about these tests are available in the supplementary material.

### Data analysis

Psychometric ‘impairment’ was defined as a score falling 1.5 standard deviations (SD) or more below the normative mean, as this is sensitive enough to detect cognitive dysfunction while maximising specificity [14].

Summary statistics were derived for cohort demographics and memory tests for the total cohort, and then for the seropositive and seronegative cohorts separately. Missing data were treated with pairwise deletion.

Memory test means were subject to correlation analyses to examine their relationship to demographic variables and are reported as Spearman’s rank correlation coefficients.

The test means of the seropositive and seronegative groups were subject to an independent *t*-test to assess for significant differences. Statistical assumptions were checked using Levene’s test (homogeneity variance) and the Shapiro–Wilk test (normality). When violated, Mann–Whitney *U* tests were reported. To assess disparities in demographic and clinical variables between seropositive and seronegative cohorts, independent *t*-tests and chi-squared analyses were conducted. Additionally, a comparison of test means and disparities in demographic and clinical variables was performed to compare anti-NMDAR ab-mediated AE versus anti-LGI1 ab-mediated AE cohorts. Furthermore, comparisons were made between anti-NMDAR ab-mediated AE and all other seropositive cases, as well as between anti-LGI1 ab-mediated AE and all other seropositive cases.

A comparison between the observed and expected outcomes was conducted using chi-squared goodness-of-fit tests. The expected outcome was determined as psychometric impairment, defined as performance 1.5 standard deviations below the normative mean. In this context, 6.7% of patients in a normative cohort would typically be classified as impaired. No multiple comparison corrections were employed in these analyses.

Psychometric patterns of impairment were determined using pattern analysis, a process involving the calculation of distinct patterns of psychometric findings through R, utilising the *confreq* package.

Pattern analysis was confined exclusively to immediate (or total) recall and free delayed recall measures, as these measures are recognised as robust indicators of memory functions. This focused approach was chosen to maintain consistency across memory paradigms, ensuring that the selected measures align with well-established metrics of memory performance.

In conducting pattern analysis, any missing data were addressed through list-wise deletion.

Analysis conducted on JASP (version 0.16.3) and R studio (version 1.2.5042). Graphs were created using GraphPad Prism (version 9.0.0) and Microsoft Excel (Version 16.66.1).

## Results

### Patient characteristics

The final sample contained 52 patients with possible AE and included 27 females and 25 males. Twenty-six (50%) were seropositive; 11 with anti-*N*-methyl-d-aspartate receptor (NMDAR) ab-mediated AE, 10 with anti-leucine-rich glioma-inactivated-1 (LGI-1) ab-mediated AE, 2 with contactin-associated protein-like 2 (CASPR-2) ab-mediated AE, and 1 with voltage-gated potassium channel complex (Unspecified; VGKC) ab-mediated AE antibodies, and 2 with another antibody. Twenty-six (50%) were seronegative. In the total cohort, 16 individuals (30.76%) underwent assessment between 6 to 12 months from their initial hospital admission. Thirteen individuals (25.00%) were assessed between 1 and 3 years after their admission, while 22 individuals (42.31%) underwent assessment between 3 and 10 years after admission. One individual (1.92%) was assessed 10 years post-admission. Demographic data is available in Table 1.

**Table 1:** Cohort demographics

Characteristics	Total cohort			Seropositive cohort			Seronegative cohort		
	Median (SD)	IQR	*n*	Median (SD)	IQR	*n*	Median (SD)	IQR	*n*
Age, years	56.50 (18.33)	31.50	52	58.5 (19.94)	31.50	26	56.00 (16.95)	33.75	26
Sex, female (*N* (%))	27 (51.92)		52	12 (46.15)		26	15 (57.69)		26
Months between symptom onset and neuropsychological assessment	29.00 (32.39)	50.00	51	46.00 (32.37)	42.75	26	17.00 (29.94)	36.00	26
Months between hospital admission and neuropsychological assessment	27.00 (31.09)	47.00	51	43.50 (30.73)	37.50	26	17.00 (29.47)	33.00	26
Months between symptom onset to hospital admission	0.00 (5.42)	1.25	51	1.00 (4.88)	6.50	26	0.00 (5.85)	1.00	26
Education, years	12.00 (2.84)	4.50	52	12.00 (2.77)	4.00	2	12.00 (2.96)	5.00	26
Telehealth, y (*N* (%))	26 (50)		52	3 (11.54)		26	2 (7.69)		26
Treatment Line, y (*N* (%))									
First line	51 (100)		51	26 (100.00)		26	25 (100)		25
Second line	27 (52.941)		51	10 (38.46)		26	17 (68.00)		25
Third line	0 (0)		51	0 (0.00)		26	0 (0.00)		25
ASM use, y (*N* (%))									
0 ASM	25 (50.00)		50	15 (57.69)		25	10 (40.00)		25
1 ASM	12 (24.00)		50	5 (19.23)		25	7 (28.00)		25
2 + ASM	13 (26.00)		50	5 (19.23)		25	8 (32.00)		25
ICU admission during main hospital admission, y (*N* (%))	22 (47.83)		46	10 (38.46)		23	12 (46.15)		24
mRS at discharge (*N* (%))									
1	9 (19.15)		47	5 (21.74)		23	4 (16.66)		24
2	18 (38.30)		47	8 (34.78)		23	10 (41.66)		24
3	17 (36.17)		47	9 (39.13)		23	8 (33.33)		24
4	3 (6.38)		47	1 (4.34)		23	2 (8.33)		24
Seropositive, y (*N* (%))			52	–	–	–	–	–	–
Anti NMDAR Ab-mediated AE	11 (42.31)		26	–	–	–	–	–	–
Anti LGI-1 Ab-mediated AE	10 (38.46)		26	–	–	–	–	–	–
Anti CASPR2 Ab-mediated AE	2 (7.69)		26	–	–	–	–	–	–
Anti VGKC Ab-mediated AE	1 (3.85)		26	–	–	–	–	–	–
Other antibodies	2 (7.69)		26	–	–	–	–	–	–

*Ab* antibody; *NMDAR N*-methyl-d-aspartate receptor; *LGI-1* anti-leucine-rich glioma-inactivated-1; *CASPR2* contactin-associated protein-like 2; *VGKC* voltage-gated potassium channel complex; *ASM* anti-seizure medication; *ICU* intensive care unit; *mRS* modified Rankin Scale

### Memory test characteristics

**Fig. 1:**
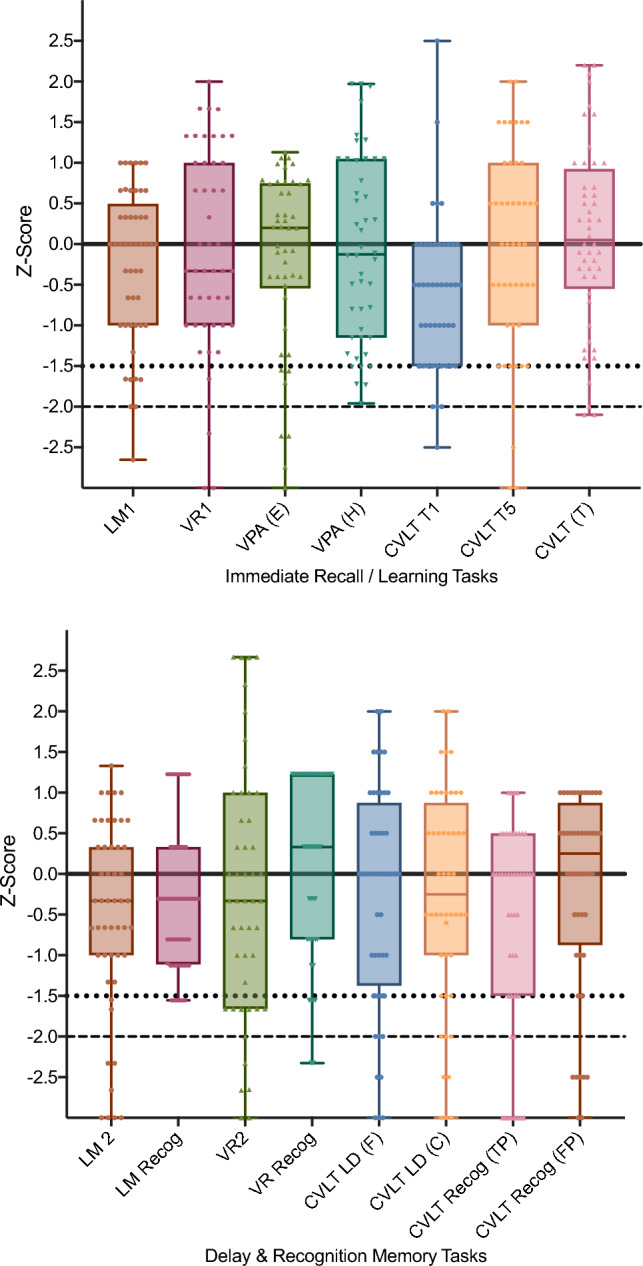
Box plot scores on memory tests for the cohort of AE patients. Normative data mean is denoted by a black line. Scores below the dotted lines are 1.5 standard deviations below the normative mean and are considered mildly impaired. Scores below the dashed line are -2.0 standard deviations below the normative mean and are considered severely impaired. *LM* logical memory; *VR* visual reproduction; *VPA* verbal paired associates; *CVLT* California verbal learning test; *E* easy pair; *H* hard pair; *T* total; *F* free recall; *C* cued recall; *TP* true positive; *FP* false positive; *LD* long delay

The characteristics of each memory test are summarised in Table 2. A visualisation of the distribution of scores across the total cohort, seropositive, and seronegative cohorts is provided in Fig. 1.

**Table 2:** Subtests characteristics for the total cohort

	Cohort *n*	*M* (*SD*)	Min	Max	*n* (%) Impaired at -1.5 SD below mean
Total cohort					
LM1	49	−0.24 (0.94)	−2.7	1	7 (14.29)
LM2	49	−0.53 (1.2)	−3.1	1.3	8 (16.33)
LM recognition	49	−0.32 (0.87)	−1.6	1.2	5 (10.20)
VR1	47	−0.11 (1.3)	−3.1	2	4 (8.51)
VR2	47	−0.21 (1.6)	−3.1	2.7	11 (23.40)
VR recognition	47	0.18 (1.1)	−2.3	1.2	6 (12.77)
VPA easy	46	−0.19 (1.2)	−4.3	1.1	6 (13.04)
VPA hard	46	−0.05 (1.1)	−2	2	5 (10.87)
CVLT T1	48	−0.64 (0.92)	−2.5	2.5	13 (27.08)
CVLT T5	48	−0.11(1.3)	−3.0	2.0	10(20.83)
CVLT total	48	0.10 (1.1)	−2.1	2.2	3 (6.25)
CVLT SD (F)	48	−0.09 (1.4)	−4	2	10 (20.83)
CVLT SD (C)	48	−0.18(1.5)	−4.5	2.0	12 (25.00)
CVLT LD (F)	48	−0.33 (1.5)	−4.5	2	12 (25.00)
CVLT LD (C)	48	−0.30(1.4)	−4.5	2.0	11 (22.92)
CVLT recognition (TP)	48	−0.82 (1.7)	−5	1	11 (22.92)
CVLT recognition (FP)	48	−0.19 (1.8)	−3.5	1	9 (18.75)

*LM* logical memory; *VR* visual reproduction; *VPA* verbal paired associates; *CVLT* California verbal learning test; *F* free recall; *C* Cued Recall; *TP* true positive; *F* false positive; *SD* short delay; *LD* long delay; *E* easy pair; *H* hard pair; *T* total

Subtest characteristics for the seropositive and seronegative cohort are available in the supplementary material (Table S1).

### Frequency of impairment on memory tests

**Fig. 2:**
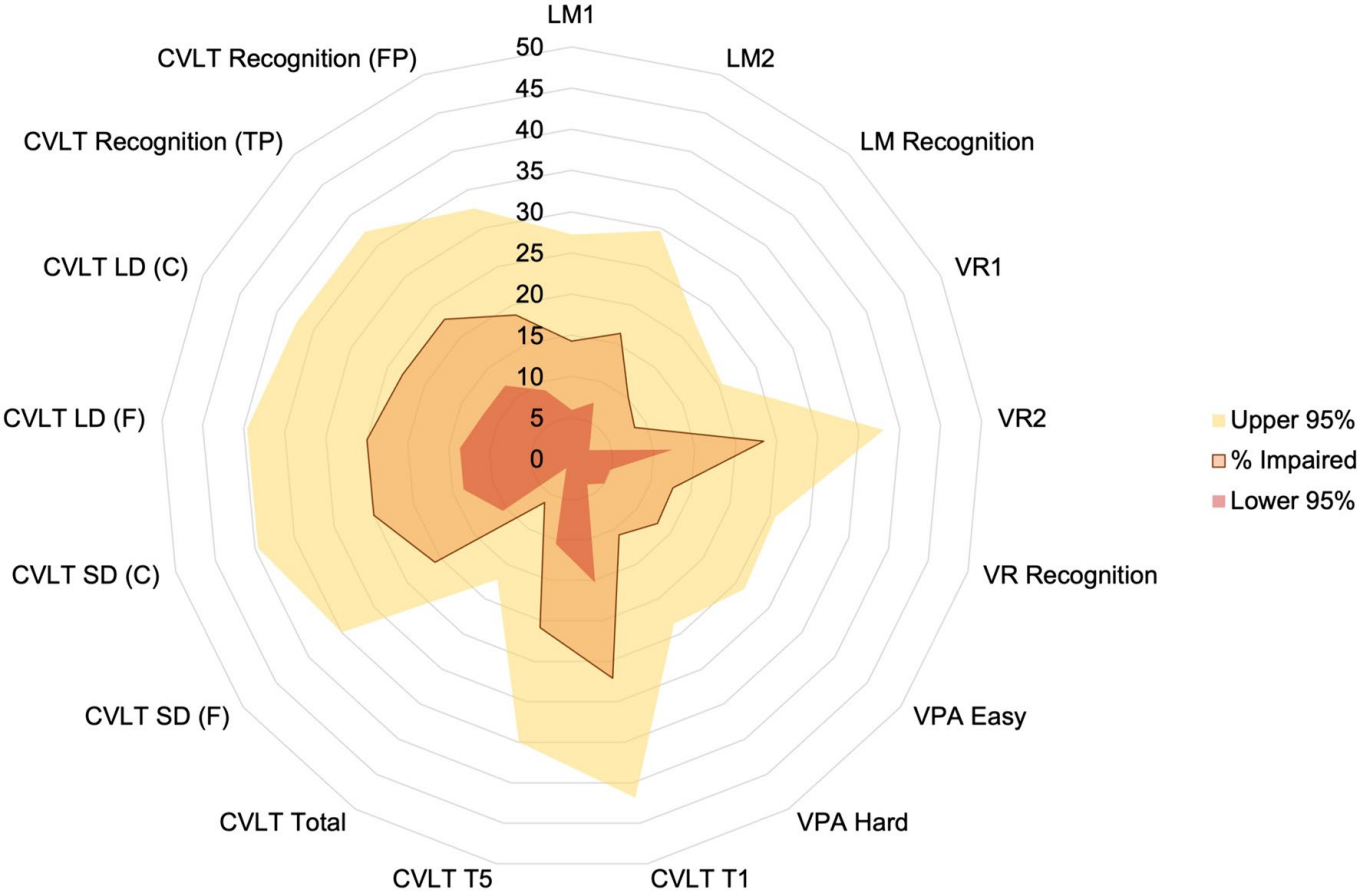
Frequency of deficits when defined at 1.5 SD below the normative mean. The pale yellow represents the upper 95% confidence interval, the dark red represents the lower 95% CI, and the orange with a dark line represents the frequency for the total cohort. *LM* logical memory; *VR* visual reproduction; *VPA* verbal paired associates; *CVLT* California verbal learning test; *F* free recall; *C* cued recall; *TP* true positive; *FP* false positive; *SD* short delay; *LD* long delay

**Fig. 3:**
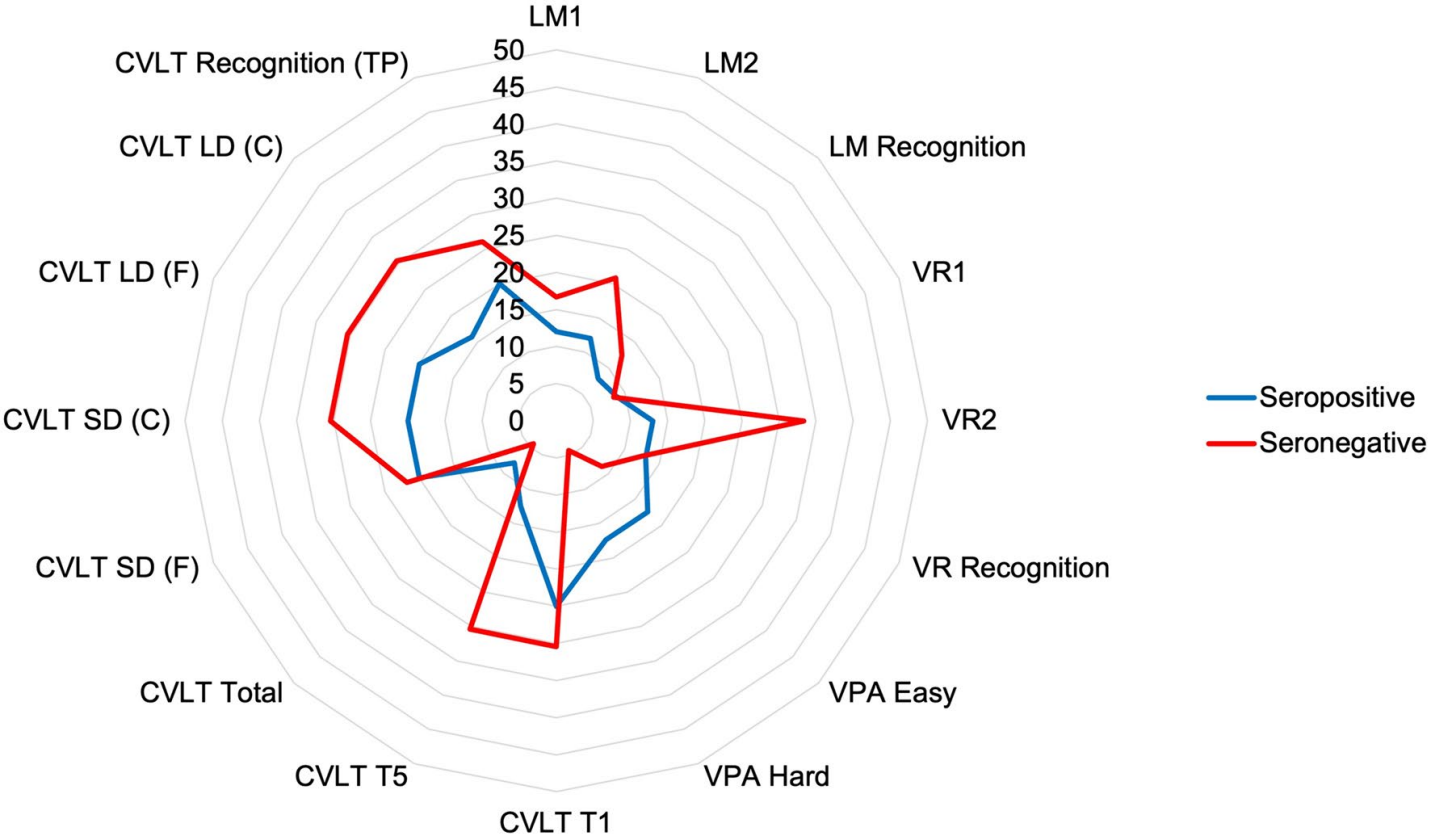
Frequency of deficits by seropositive and seronegative when at 1.5 SD below the normative mean. The blue line represents the seropositive cohort, whilst the red line represents the seronegative cohort. *LM* logical memory; *VR* visual reproduction; *VPA* verbal paired associates; *CVLT* California verbal learning test; *F* free recall; *C* cued recall; *TP* true positive; *FP* false positive; *SD* short delay; *LD* long delay

When impairment was defined at 1.5 standard deviations below the normative mean, the frequency of impairments on memory tests is available in the supplementary material (Table S2). Figures 2 and 3 visualise the percentage of impairments for the total cohort and then for the seronegative and seropositive cohorts, respectively.

### Chi-squared goodness of fit tests

For the total cohort, Chi-squared goodness of fit tests were conducted to compare the actual number of impaired patients to the expected number of impaired patients under a normal distribution, where impaired is defined at 1.5 standard deviations below the normative mean. This revealed significant differences for LM1 (χ^2^(1, *N* = 49) = 5.68, *p* = 0.017), LM2 (χ^2^(1, *N* = 49) = 8.87, *p* = 0.003), VR2 (χ^2^(1, *N* = 47) = 22.78, *p* < 0.00001), CVLT T1 (χ^2^(1, *N* = 48) = 35.61, *p* < 0.00001), CVLT T5 (χ^2^(1, *N* = 48) = 17.45, *p* = 0.00003), CVLT SD (F) (χ^2^(1, *N* = 48) = 17.42, *p* = 0.00003), CVLT SD (C) (χ^2^(1, *N* = 48) = 28.8, *p* < 0.00001), CVLT LD (F) (χ^2^(1, *N* = 48) = 28.8, *p* < 0.00001), CVLT LD (C) (χ^2^(1, *N* = 48) = 22.76, *p* < 0.00001), CVLT TP (χ^2^(1, *N* = 48) = 22.76, *p* < 0.00001) and CVLT FP (χ^2^(1, *N* = 48) = 12.8, *p* = 0.0035). There were no significant differences for LM Recog, VR1, VR Recog, VPA (E), VPA (H), and CVLT (T).

Table 3 presents the chi-squared goodness of fit tests for the seropositive and seronegative groups.

**Table 3:** Chi-squared goodness of fit tests for seropositive and seronegative groups

	${\chi }^{2}$	*n*	*p*
Seropositive			
LM1	0.54	25	0.46
LM2	0.54	25	0.46
LM recognition	0.00	25	1.00
VR1	0.00	23	1.00
VR2	0.55	23	0.46
VR recognition	0.55	23	0.46
VPA easy	2.19	23	0.14
VPA hard	2.19	23	0.14
CVLT T1	8.73	24	0.003*
CVLT T5	0.55	24	0.46
CVLT total	0.00	25	1.00
CVLT SD (F)	4.89	25	0.03*
CVLT SD (C)	4.89	25	0.03*
CVLT LD (F)	4.89	25	0.03*
CVLT LD (C)	2.17	25	0.14
CVLT recognition (TP)	4.89	25	0.03*
CVLT recognition (FP)	4.89	25	0.03*
Seronegative			
LM1	2.18	24	0.14
LM2	4.91	24	0.03*
LM recognition	0.55	24	0.46
VR1	0.00	24	1.00
VR2	19.64	24	< 0.00001**
VR recognition	0.55	24	0.46
VPA easy	0	23	1
VPA hard	0.55	23	0.46
CVLT T1	13.69	23	0.0002**
CVLT T5	13.69	23	0.0002**
CVLT total	0.55	23	0.46
CVLT SD (F)	4.93	23	0.03
CVLT SD (C)	13.69	23	0.0002**
CVLT LD (F)	13.69	23	0.0002**
CVLT LD (C)	13.69	23	0.0002**
CVLT recognition (TP)	8.76	23	0.003*
CVLT recognition (FP)	2.19	23	0.14

*p < 0.05, **p < 0.001

*LM* logical memory; *VR* visual reproduction; *VPA* verbal paired associates; *CVLT* California verbal learning test; *F* free recall; *C* cued recall; *TP* true positive; *F* false positive; *SD* short delay; *LD* long delay; *E* easy pair; *H* hard pair; *T* total

### Pattern analysis

Pattern analysis encompassed both the learning/immediate tests and the tests for free delayed recall of the material. Forty-two patients were eligible for inclusion in the analysis as they had successfully completed all the tests incorporated in the pattern analysis. Seventeen distinct patterns were identified and are presented in Table 4. Table S7 presents the patterns for the seropositive and seronegative groups. The prevailing pattern observed was intact memory outcomes, with 52.76% of patients exhibiting this particular pattern. Notably, 47.24% of patients experienced impairment in at least one test.

**Table 4:** Patterns of memory outcomes

Pattern of psychometric impairment	*n*	%
Intact	23	54.76
Impaired CVLT LD (F)	2	4.76
Impaired V2, CVLT LD (F)	2	4.76
Impaired LM1, LM2	2	4.76
Impaired VPA (H)	1	2.38
Impaired VPA (E)	1	2.38
Impaired VR2	1	2.38
Impaired VR2, CVLT (T), CVLT LD (F)	1	2.38
Impaired VR2, VPA (H)	1	2.38
Impaired VR2, VPA (H), CVLT LD (F)	1	2.38
Impaired VR2, VPA (E)	1	2.38
Impaired VR1	1	2.38
Impaired VR1, VR2, VPA (E),	1	2.38
Impaired LM1, LM2, CVLT LD (F)	1	2.38
Impaired LM1, LM2, VPA (E), VPA (H)	1	2.38
Impaired LM1, LM2, VPA (E), VPA (H), CVLT LD (F)	1	2.38
Impaired LM1, LM2, VR1, VR2, VPA (E), CVLT (T), CVLT LD (F)	1	2.38
	42	

*LM* logical memory; *VR* visual reproduction; *VPA* verbal paired associates; *CVLT* California verbal learning test; *F* free recall; *LD* long delay; *E* easy pair; *H* hard pair; *T* total

### Independent *T*-tests

#### Seropositive vs seronegative

There was no evidence of differences across all memory tests between patients classified as seropositive and those classified as seronegative (supplementary data Table S3) using independent samples *t*-tests.

Seropositive patients (*M* = 49.62, SD = 32.37) had a significantly longer time between symptom onset and assessment compared to seronegative patients (*M* = 30.36, SD = 29.94) (U [*N*_seropositive_ = 26, *N*_seronegative_ = 25] = 188.50, *p* = 0.010). Seropositive patients (*M* = 46.11, SD = 30.73) had longer between main hospital admission and assessment than seronegative patients (*M* = 28.68, SD = 9.47) (U [*N*_seropositive_ = 26, *N*_seronegative_ = 25] = 202.00, *p* = 0.021). Seropositive patients (*M* = 3.58, SD = 4.88) had a longer time between symptom onset and admission than seronegative patients (*M* = 1.61, SD = 5.85) (U [*N*_seropositive_ = 26, *N*_seronegative_ = 25] = 224.00, *p* = 0.024). Seronegative patients were more likely to have had second-line therapies than seropositive patients (χ^2^ (1,51) = 4.46, *p* = 0.035).

### Anti-NMDAR ab-mediated AE vs anti-LGI1 ab-mediated AE

Results from an independent samples *t*-test indicated that patients diagnosed with anti-NMDAR ab-mediated AE patients (*M* = 0.25, SD = 0.87, *N* = 11) scored higher on LM2 than patients diagnosed with anti-LGI1 ab-mediated AE (*M* = −0.76, SD = 1.31, *N* = 9), *t* (18) = 2.30, *p* = 0.03). There were no significant differences for the remaining memory tests (supplementary data table S4). There was no evidence of differences in demographic or clinical data between these two groups.

### Anti-NMDAR ab-mediated AE versus all other seropositive AE cases

Results from an independent sample t-test indicated that patients diagnosed with anti-NMDAR ab-mediated AE patients (*M* = 0.25, SD = 0.87, *N* = 11) scored higher on LM2 than all other patients who were seropositive (*M* = −0.83, SD = 1.08, *N* = 14), *t*(23) = −2.71, *p* = 0.03). There was no evidence of differences between the rest of the memory tests (supplementary data table S4). There was no evidence of differences in demographic or clinical data between these two groups.

### Anti-LGI-1 ab-mediated AE versus all other seropositive AE cases

There was no evidence of differences across all memory tests between patients diagnosed with anti-LGI1 ab-mediated AE and all other seropositive patients (supplementary data Table S6). There was no evidence of differences in demographic or clinical data between these two groups.

### Correlations between demographic and clinical variables and memory tests

There was a significant correlation between time between symptom onset and admission and VR Recog (*r*(45) = 0.32,* p* = 0.03), VPA (H) (*r*(44) = 0.38, *p* = 0.01) and CVLT T1 (*r*(45) = 0.33, *p* = 0.03). There were significant correlations between mRS at discharge and a number of variables from the CVLT: CVLT T5 (*r*(45) = −0.33, *p* = 0.03), CVLT T (*r*(45) = −032, *p* = 0.05), CVLT SD (F) (*r*(45) = −0.43, *p* = 0.004), CVLT SD (C) (*r*(45) = −0.31, *p* = 0.04), CVLT LD (F) (*r*(45) = −0.38, *p* = 0.01), CVLT LD (C) (*r*(45) = −0.39, *p* = 0.009), and CVLT Recog (FP) (*r*(45) = −0.34, *p* = 0.03).

## Discussion

Characterising the extent and type of memory impairment in AE requires detailed and systematic assessment. We employed a comprehensive set of memory tests in a prospectively recruited group of AE patients who met the criteria for (at a minimum) possible AE. The cohort consisted of both seropositive and seronegative patients. We sought to characterise memory performances by comparing them to normative data. Four key findings emerged from this study. First, on average, there was no significant impairment on memory measures for the cohort and across the seropositive and seronegative groups. Second, frequency data indicated that approximately one-fifth to a quarter of patients performed below normative data on several memory measures. Third, the anti-LGI1 ab-mediated AE group scored lower on a logico-semantic memory delayed test than the anti-NMDAR ab-mediated group. Fourth, in the pattern analysis, it was found that 54.76% of patients exhibited intact memory measures. For those with psychometric impairment, 16 distinct patterns were identified.

On average, none of the memory measures for the total cohort, the seropositive nor seronegative group, were significantly below the normative mean. Despite these overall findings, frequency analysis demonstrated impairments on memory measures observed in the cohort—where in the total cohort, approximately one-fifth of the patients had impairments on delayed recall of visual material, acquisition of a list of words, and all measures of recall for this list. The frequency of these observed deficits was beyond what would be expected for a normative cohort. When explored by serostatus, at least one-fifth (up to over a quarter on some tests) of the patients were impaired on those same list-learning measures for both the seropositive and seronegative. Additionally, the seronegative group had at least one-fifth of patients impaired on the delayed recall of the logicosemantic test and the delayed recall of visual material.

Pattern analysis allows for identifying patterns in memory performances across a cohort of individuals. This allows the identification of commonalities and variations in cognitive profiles within a specific population, enabling a move beyond individual differences and individual cognitive measures and focusing on a more comprehensive understanding of cognitive outcomes. The current study’s pattern analysis results highlight notable variability in memory outcomes. Fifty-four percent of patients demonstrated intact psychometric performances. However, for patients exhibiting psychometric impairment, there was substantial variability in the specific memory measures affected. This emphasises the importance of conducting a thorough memory assessment in AE patients, using a diverse range of measures to accurately characterise memory, given the diverse ways various memory measures were implicated. Further, the observed variability underscores the need for a comprehensive approach to understanding memory outcomes in chronic AE.

It is crucial to acknowledge that pattern analysis, as employed in this study, comes with the prerequisite that patients must complete all the measures included in the analysis. Unfortunately, this poses a challenge. Due to cognitive challenges, a number of the recruited patients were unable to complete the full battery of tests. Consequently, this pattern analysis is unlikely to underestimate the number of patients with significant memory or global cognitive impairments, as those with severe cognitive limitations could not fulfil the criteria for inclusion in the pattern analysis. This is particularly of note as previous research has demonstrated some patients experience significant memory impairments following AE. Specifically, there is broad consensus that LGI1 ab-mediated AE patients can have significant primary memory impairments [2, 4, 20–22]. These significant memory impairments have been associated with the integrity of neuroanatomical memory structures in patients with anti-LGI1 ab-mediated AE [22]. However, given that there are mixed reports regarding memory outcomes in anti-LGI1 ab-mediated AE, if the literature is considered wholly, it could be suggested that there may be two distinct memory profiles in anti-LGI1 ab-mediated AE patients [20]. The first profile could be defined by primary memory impairment and may be associated with the reported reduced integrity of the hippocampus and its structures. A second profile could theoretically be characterised by memory secondarily impacted by attentional variability and/or executive dysfunction [20]. Further investigation, with larger sample sizes and a focus on linking these hypothesised profiles to neuroanatomical and cognitive data, is essential to ascertain the validity of this hypothesis and determine whether distinct memory profiles truly exist in anti-LGI1 ab-mediated AE patients and whether this can be extrapolated to other ab-mediated AE’s. However, as noted in the introduction, memory deficits may coincide with impairments in other cognitive domains, collectively contributing to functional memory difficulties. This intersection is particularly significant given the evidence of various cognitive deficits following autoimmune encephalitis [5, 7, 9, 10, 23, 24]. Additionally, psychological or psychiatric factors, such as anxiety, depression, and stress, as well as ongoing seizure activity can further exacerbate memory impairments, highlighting the need for a comprehensive assessment approach that considers these influences in AE patients, Future research allowing the integration of these factors may offer a clearer pattern of cognitive outcomes, elucidating the interplay between these domains and their functional presentations.

Concerning relationships between clinical and demographic data, of specific note were the significant differences between seropositive and seronegative groups and the time between (a) admission to assessment (i.e. recruitment), (b) symptom onset to assessment, and (c) symptom onset to admission. Here, seropositive were more likely to have longer times between these time points. The effect on the time between both admission and symptom onset to assessment may be secondary to recruitment bias. As the patients were recruited through outpatient neurology clinics, it may be that the seropositive patients have longer follow-ups compared to seronegative patients; however, the reason for this is unclear.

Concerning the time between symptom onset and hospital admission, the reason is unknown as to why seropositive patients in this cohort had a longer time to admission than the seronegative cohort. There is, however, evidence to suggest that delayed treatment can result in poorer cognitive outcomes [25]. Notably, the three memory measures correlated with this variable are heterogeneous—a visual recognition test, verbal arbitrarily associated pair learning, and the first trial of a list learning test. As each memory variable reflects significantly different memory paradigms, they cohesively do not suggest anything specific regarding memory outcomes at this time and thus require further investigation. In addition, there was notable variability in the time elapsed from diagnosis to testing underscores the complexity of assessing cognitive function in individuals with neurological conditions. For example, given the dynamic nature of cognition in patients who develop seizures after AE, this variability can significantly influence the broader characterisation of cognitive functioning. Further, longer intervals between diagnosis and testing may introduce confounding variables such as changes in medication regimen, comorbidities, or psychosocial factors, all of which can influence cognitive outcomes. Consequently, this variability introduces a significant confounding factor that warrants careful consideration in future research characterising memory functions in AE.

### Limitations

While the sample size for this study represents a large proportion of patients with this disease in Victoria, Australia, it is limited. The application of several memory paradigms is a first in AE and provides a clearer picture of memory outcomes in this population. Despite these factors, there are limitations. First, the study results are based on psychometric scores only, not clinical diagnosis. This limits the ability to draw definitive conclusions about whether the poor memory scores reported here reflect a primary memory impairment. Further, factors influencing memory performances in neuropsychological testing, including psychopathology and fatigue, were not investigated.

Second, the investigation is cross-sectional, limiting any causal relationship that can be concluded. Consequently, we cannot directly determine whether the ‘normal’ memory performances observed in the cohort indicate recovery from memory compromise in the acute state or the absence of memory disruption on average during AE. This raises questions as to whether the cross-sectional results presented in our study represent recovery or a stable memory profile across the disease period. Additionally, mRS scores, which are used to measure disability, may also be influenced by this limitation, as changes in mRS could reflect both recovery and ongoing impairments. Future research should include acute patients to address this limitation and gain a more comprehensive understanding of memory outcomes and their trajectory in AE. Third, accelerated long-term forgetting and retrograde amnesia were not investigated, with this study utilising memory tests with a typical 20 to 30-min delay interval. However, previous literature has highlighted the importance of examining these memory constructs in limbic encephalitis [26, 27]. Therefore, future investigations should incorporate measures of accelerated long-term forgetting and/or retrograde amnesia to explore further memory dysfunction associated with AE. Fourth, no correction was applied for multiple comparisons due to small sample sizes, leading to an increased risk of false positives and the potential for obtaining statistically significant outcomes through random chance. This can influence the validity and generalisability of the findings, and caution should be taken when interpreting results. Finally, the absence of EEG and imaging data limits the generalisability of our findings. Consequently, our findings should be approached with caution, recognising the need for additional neurophysiological data to provide a comprehensive understanding of cognitive processes underlying memory outcomes.

## Conclusion

The study comprehensively and prospectively examined memory deficits in a cohort of patients with AE. The in-depth exploration of memory sheds light on the clinical implications of our findings, indicating that while memory is not always a psychometric deficit after AE, there is a subgroup of patients for which psychometric scores indicated poor memory performances. Overall, the studies emphasised the clinical diversity in memory outcomes within the AE population and highlighted the need for further research to confirm and expand on these findings. Future investigations involving larger sample sizes and incorporating neuroimaging are warranted to validate these memory profiles and their underlying mechanisms.

### Supplementary Information

Below is the link to the electronic supplementary material.

Supplementary file1 (DOCX 38 KB)

## Data Availability

The data supporting this study's findings are available from the corresponding author upon reasonable request.
