# Supplementary material for: Memory function in autoimmune encephalitis: a cross-sectional prospective study utilising multiple memory paradigms
Source: J Neurol. 2024 Jun 25;271(8):5610–21. doi: 10.1007/s00415-024-12520-z (PMC11319369; doi:10.1007/s00415-024-12520-z)
Supplement: Supplementary file 1 — Supplementary file1 (DOCX 38 KB) [file 415_2024_12520_MOESM1_ESM.docx]

**Supplementary Methods**

**Materials**

***California Verbal Learning Test – 2^nd^ Edition (Standard Form)***(12)

The California Verbal Learning Test – 2^nd^ Edition (CVLT) measures verbally mediated learning and delayed recall. Two 16-item word lists are read aloud, which are made up of four semantic categories, with each category containing four words. The subject is asked to recall the first list (List A) after each of the five exposures (T1 to T5). An interference trial (List B) is administered. This is followed by a free recall of List A (CVLT SD (F)) and a cued recall of List A by semantic categories (CVLT SD (C)). After a 20–25-minute delay, free (CVLT LD (F)) and cued recall (CVLT LD (C)), subjects are then provided with a yes/no recognition trial. The number of true-positives (CVLT Recognition (TP)) and false-positives (CVLT Recognition (FP)) were recorded. All the raw scores were standardised to z-scores per the test manual.

***Wechsler Memory Scale – 4^th^ Edition***(13)

***Logical Memory***

Immediate and delayed logicosemantic memory were measured by the Logical Memory (LM) subtest test: immediate (LM-1), delayed recall (LM-2), and recognition (LM Recog) test from the Wechsler Memory Scale 4th edition (WMS-IV). In LM-1, subjects are asked to recall details of two prose passages immediately. After a 20 to 30-minute delay, subjects are asked to recall the details of these two passages (LM-2). In LM Recognition, subjects are provided with yes/no questions on the two proses.

***Visual Reproduction***

Visual immediate and delayed memory were measured by the Visual Reproduction (VR) subtest - immediate (VR-1), delayed recall (VR-2), and recognition (VR Recog) test from the WMS-IV. In VR-1, subjects are provided with five designs presented separately. Items 1 to 3 consist of one design, and items 4 to 5 with each with two designs. After 20-30 minutes, subjects are asked to re-draw the designs without exposure (VR2). This is followed by a recognition trial in which subjects are asked to choose which of six designs on a display matches the original design shown.

For all raw scores derived, the WMS-IV manual provides standardised scores through age-matched normative data for each of the tests within the subtests of the WMS-IV. For LM1, LM2, VR1 and VR2, the standard scores were converted to z-scores. For LM Recognition and VR recognition, the scores produced are cumulative percentage ranges. The median was derived, and this score was converted to a z-score.

***Wechsler Memory Scale – 1^st^ Edition***(14)

***Verbal Paired Associates Subtest***

The Verbal Paired Associates (VPA) subtest from the Wechsler Memory Scale-1 (WMS-1) measures verbal arbitrary and semantic associative learning. The subject is read two types of word pairs – arbitrarily associated pairs (‘hard pairs’ [VPA (H)], e.g., school-grocery) and semantically related pairs (‘easy pairs’ [VPA (E)], e.g., fruit-apple). These word pairs are presented as a list and learned over three exposures. The sum of trials 1 to 3 are summated by the type of pair (VPA (H); VPA (E)) and converted into z-scores using normative data.

**Supplementary Data**

| **Table S1.** | | | | | | |
| --- | --- | --- | --- | --- | --- | --- |
| **Seropositive** | | | | | | |
| LM1 | 25 | -0.07 (0.94) | | -2 | 1 | 3 (12.00) |
| LM2 | 25 | -0.35 (1.1) | | -3 | 1.3 | 3 (12.00) |
| LM Recognition | 25 | -0.17 (0.89) | | -1.6 | 1.2 | 2 (8.00) |
| VR1 | 23 | -0.06 (1.2) | | -3 | 1.7 | 2 (8.70) |
| VR2 | 23 | 0.06 (1.5) | | -3.1 | 2.7 | 3 (13.04) |
| VR Recognition | 23 | 0.23 (1.2) | | -2.3 | 1.2 | 3 (13.04) |
| VPA Easy | 23 | -0.45 (1.3) | | -4.3 | 1.1 | 4 (17.39) |
| VPA Hard | 23 | -0.15 (1.3) | | -2 | 2 | 4 (17.39) |
| CVLT T1 | 24 | -0.42 (1.1) | | -2.5 | 2.5 | 6 (25.00) |
| CVLT T5 | 24 | 0.04 (1.2) | | -3 | 2 | 3 (12.50) |
| CVLT Total | 25 | 0.09 (1.2) | | -2.1 | 2.2 | 2 (8.00) |
| CVLT SD (F) | 25 | -0.1 (1.3) | | -2.5 | 2 | 5 (20.00) |
| CVLT SD (C) | 25 | 0.00 (1.3) | | -3 | 2 | 5 (20.00) |
| CVLT LD (F) | 25 | -0.08 (1.3) | | -2.5 | 2 | 5 (20.00) |
| CVLT LD (C) | 25 | -0.1 (1.2) | | -3 | 2 | 4 (16.00) |
| CVLT Recognition (TP) | 25 | -0.84 (1.6) | | -5 | 1 | 5 (20.00) |
| CVLT Recognition (FP) | 25 | -0.1 (1.2) | | -3 | 1 | 5 (20.00) |
| **Seronegative** | | | | | | |
| LM1 | 24 | -0.43 (0.92) | | -2.7 | 0.67 | 4 (16.67) |
| LM2 | 24 | -0.71 (1.2) | | -3.1 | 1 | 5 (20.83) |
| LM Recognition | 24 | -0.47 (0.84) | | -1.6 | 1.2 | 3 (12.50) |
| VR1 | 24 | -0.17 (1.3) | | -3.1 | 2 | 2 (8.33) |
| VR2 | 24 | -0.47 (1.6) | | -3 | 2.7 | 8 (33.33) |
| VR Recognition | 24 | 0.13 (1.1) | | -1.6 | 1.2 | 3 (12.50) |
| VPA Easy | 23 | 0.07 (0.96) | | -2.4 | 1.1 | 2 (8.70) |
| VPA Hard | 23 | 0.07 (0.96) | | -1.7 | 1.8 | 1 (4.35) |
| CVLT T1 | 23 | -0.87 (0.68) | -2.0 | | 0.0 | 7 (30.43) |
| CVLT T5 | 23 | -0.26 (1.3) | | -3 | 2 | 7 (30.43) |
| CVLT Total | 23 | 0.10 (1.1) | | -2.1 | 2.2 | 1 (4.35) |
| CVLT SD (F) | 23 | -0.09 (1.5) | | -4 | 2 | 5 (21.74) |
| CVLT SD (C) | 23 | -0.37 (1.6) | | -4.5 | 1.5 | 7 (30.43) |
| CVLT LD (F) | 23 | -0.61 (1.7) | | -4.5 | 2 | 7 (30.43) |
| CVLT LD (C) | 23 | -0.52 (1.6) | | -4.5 | 2 | 7 (30.43) |
| CVLT Recognition (TP) | 23 | -0.8 (1.9) | | -5 | 1 | 6 (26.09) |
| CVLT Recognition (FP) | 23 | -0.28 (1.3) | | -3.5 | 1 | 4 (17.39) |

*Abb: LM = Logical Memory; VR = Visual Reproduction; VPA = Verbal Paired Associates; CVLT = California Verbal Learning Test; F = Free Recall; C = Cued Recall; TP = True Positive; F = False Positive; SD = Short Delay; LD = Long Delay; E = Easy Pair; H = Hard Pair; T = Total*

| **Table S2.** | Impaired (%) | 95% CI (Lower) | 95% CI (Upper) |
| --- | --- | --- | --- |
| **Total Cohort** | | | |
| LM1 | 14.29 | 5.94 | 27.24 |
| LM2 | 16.33 | 7.32 | 29.66 |
| LM Recognition | 10.20 | 3.40 | 22.23 |
| VR1 | 8.51 | 2.37 | 20.38 |
| VR2 | 23.40 | 12.30 | 38.03 |
| VR Recognition | 12.77 | 4.83 | 25.74 |
| VPA Easy | 13.04 | 4.94 | 26.26 |
| VPA Hard | 10.87 | 3.62 | 23.57 |
| CVLT T1 | 27.08 | 15.28 | 41.85 |
| CVLT T5 | 20.83 | 10.47 | 34.99 |
| CVLT Total | 6.25 | 1.31 | 17.20 |
| CVLT SD (F) | 20.83 | 10.47 | 34.99 |
| CVLT SD (C) | 25.00 | 13.64 | 39.60 |
| CVLT LD (F) | 25.00 | 13.64 | 39.60 |
| CVLT LD (C) | 22.92 | 12.03 | 37.31 |
| CVLT Recognition (TP) | 22.92 | 12.03 | 37.31 |
| CVLT Recognition (FP) | 18.75 | 8.95 | 32.63 |
| **Seropositive** | | | |
| LM1 | 12.00 | 2.55 | 31.22 |
| LM2 | 12.00 | 2.55 | 31.22 |
| LM Recognition | 8.00 | 0.98 | 26.03 |
| VR1 | 8.70 | 1.07 | 28.04 |
| VR2 | 13.04 | 2.77 | 33.59 |
| VR Recognition | 13.04 | 2.77 | 33.59 |
| VPA Easy | 17.39 | 4.95 | 38.78 |
| VPA Hard | 17.39 | 4.95 | 38.78 |
| CVLT T1 | 25.00 | 9.77 | 46.71 |
| CVLT T5 | 12.50 | 2.66 | 32.36 |
| CVLT Total | 8.00 | 0.98 | 26.03 |
| CVLT SD (F) | 20.00 | 6.83 | 40.70 |
| CVLT SD (C) | 20.00 | 6.83 | 40.70 |
| CVLT LD (F) | 20.00 | 6.83 | 40.70 |
| CVLT LD (C) | 16.00 | 4.54 | 36.08 |
| CVLT Recognition (TP) | 20.00 | 6.83 | 40.70 |
| CVLT Recognition (FP) | 20.00 | 6.83 | 40.70 |
| **Seronegative** | | | |
| LM1 | 16.67 | 4.74 | 37.38 |
| LM2 | 20.83 | 7.13 | 42.15 |
| LM Recognition | 12.50 | 2.66 | 32.36 |
| VR1 | 8.33 | 1.03 | 27.00 |
| VR2 | 33.33 | 15.63 | 55.32 |
| VR Recognition | 12.50 | 2.66 | 32.36 |
| VPA Easy | 8.70 | 1.07 | 28.04 |
| VPA Hard | 4.35 | 0.11 | 21.95 |
| CVLT T1 | 30.43 | 13.21 | 52.92 |
| CVLT T5 | 30.43 | 13.21 | 52.92 |
| CVLT Total | 4.35 | 0.11 | 21.95 |
| CVLT SD (F) | 21.74 | 7.46 | 43.70 |
| CVLT SD (C) | 30.43 | 13.21 | 52.92 |
| CVLT LD (F) | 30.43 | 13.21 | 52.92 |
| CVLT LD (C) | 30.43 | 13.21 | 52.92 |
| CVLT Recognition (TP) | 26.09 | 10.23 | 48.41 |
| CVLT Recognition (FP) | 17.39 | 4.95 | 38.78 |

*Abb: LM = Logical Memory; VR = Visual Reproduction; VPA = Verbal Paired Associates; CVLT = California Verbal Learning Test; F = Free Recall; C = Cued Recall; TP = True Positive; F = False Positive; SD = Short Delay; LD = Long Delay*

| **Table S3. Seropositive vs. Seronegative** | | | | | | |
| --- | --- | --- | --- | --- | --- | --- |
|  | **Test** | **Statistic** | **df** | | **p** | |
| LM1 | Mann-Whitney | 230.000 |  | 0.161 | |  |
| LM2 | Student | -1.057 | 47.000 | 0.296 | |  |
| LM Recognition | Mann-Whitney | 237.000 |  | 0.203 | |  |
| VR1 | Student | -0.301 | 45.000 | 0.765 | |  |
| VR2 | Student | -1.164 | 45.000 | 0.250 | |  |
| VR Recognition | Mann-Whitney | 261.500 |  | 0.757 | |  |
| VPA Easy | Mann-Whitney | 343.000 |  | 0.086 | |  |
| VPA Hard | Mann-Whitney | 301.500 |  | 0.422 | |  |
| CVLT T1 | Student | -1.713 | 45.000 | 0.094 | |  |
| CVLT T5 | Student | -0.815 | 45.000 | 0.420 | |  |
| CVLT Total | Student | 0.024 | 46.000 | 0.981 | |  |
| CVLT SD (F) | Student | 0.033 | 46.000 | 0.974 | |  |
| CVLT SD (C) | Student | -0.874 | 46.000 | 0.387 | |  |
| CVLT LD (F) | Student | -1.245 | 46.000 | 0.219 | |  |
| CVLT LD (C) | Student | -1.021 | 46.000 | 0.313 | |  |
| CVLT Recognition (TP) | Mann-Whitney | 311.500 |  | 0.622 | |  |
| CVLT Recognition (FP) | Mann-Whitney | 262.500 |  | 0.606 | |  |

| **Table S4. Anti-NMDAR vs. Anti-LGI** | | | | |
| --- | --- | --- | --- | --- |
|  | **Test** | **Statistic** | **df** | **p** |
| LM1 | Mann-Whitney | 61.000 |  | 0.394 |
| LM2 | Student | 2.300 | 18 | 0.034 |
| LM Recognition | Student | -0.503 | 18 | 0.621 |
| VR1 | Student | -0.988 | 16 | 0.338 |
| VR2 | Student | 0.191 | 16 | 0.851 |
| VR Recognition | Mann-Whitney | 39.000 |  | 0.963 |
| VPA Easy | Mann-Whitney | 49.500 |  | 0.423 |
| VPA Hard | Student | 0.262 | 16 | 0.797 |
| CVLT T1 | Student | 0.099 | 17 | 0.922 |
| CVLT T5 | Student | 1.383 | 17 | 0.185 |
| CVLT Total | Student | 0.577 | 18 | 0.571 |
| CVLT SD (F) | Student | 0.934 | 18 | 0.363 |
| CVLT SD (C) | Student | 0.867 | 18 | 0.397 |
| CVLT LD (F) | Student | 0.996 | 18 | 0.332 |
| CVLT LD (C) | Student | 0.351 | 18 | 0.730 |
| CVLT Recognition (TP) | Mann-Whitney | 44.500 |  | 0.700 |
| CVLT Recognition (FP) | Student | 1.897 | 18 | 0.074 |

| **Table S5. Anti-NMDAR vs. All other seropositive** | | | | |
| --- | --- | --- | --- | --- |
|  | **Test** | **Statistic** | **df** | **p** |
| LM1 | Welch | -1.734 | 22.836 | 0.096 |
| LM2 | Student | -2.711 | 23.000 | 0.012 |
| LM Recognition | Student | 0.702 | 23.000 | 0.490 |
| VR1 | Student | 0.608 | 21.000 | 0.550 |
| VR2 | Student | -0.220 | 21.000 | 0.828 |
| VR Recognition | Mann-Whitney | 62.500 |  | 0.897 |
| VPA Easy | Mann-Whitney | 56.500 |  | 0.619 |
| VPA Hard | Mann-Whitney | 49.500 |  | 0.351 |
| CVLT T1 | Student | 0.095 | 22.000 | 0.925 |
| CVLT T5 | Student | -1.857 | 22.000 | 0.077 |
| CVLT Total | Student | -0.849 | 23.000 | 0.405 |
| CVLT SD (F) | Student | -1.290 | 23.000 | 0.210 |
| CVLT SD (C) | Student | -0.941 | 23.000 | 0.357 |
| CVLT LD (F) | Student | -1.055 | 23.000 | 0.303 |
| CVLT LD (C) | Student | -0.527 | 23.000 | 0.603 |
| CVLT Recognition (TP) | Mann-Whitney | 72.500 |  | 0.910 |
| CVLT Recognition (FP) | Mann-Whitney | 101.000 |  | 0.148 |

| **Table S6. Anti-LGI1 vs. All other seropositive** | | | | |
| --- | --- | --- | --- | --- |
|  | **Test** | **Statistic** | **df** | **p** |
| LM1 | Mann-Whitney | 64.000 |  | 0.837 |
| LM2 | Student | 1.223 | 23 | 0.234 |
| LM Recognition | Student | -0.669 | 23 | 0.510 |
| VR1 | Student | -0.909 | 21 | 0.374 |
| VR2 | Student | 0.444 | 21 | 0.662 |
| VR Recognition | Mann-Whitney | 52.500 |  | 0.835 |
| VPA Easy | Mann-Whitney | 73.000 |  | 0.419 |
| VPA Hard | Student | -0.181 | 21 | 0.858 |
| CVLT T1 | Student | 0.480 | 22 | 0.636 |
| CVLT T5 | Mann-Whitney | 75.000 |  | 0.673 |
| CVLT Total | Student | 0.599 | 23 | 0.555 |
| CVLT SD (F) | Student | 0.351 | 23 | 0.729 |
| CVLT SD (C) | Student | 0.961 | 23 | 0.347 |
| CVLT LD (F) | Student | 0.901 | 23 | 0.377 |
| CVLT LD (C) | Student | 0.371 | 23 | 0.714 |
| CVLT Recognition (TP) | Mann-Whitney | 63.500 |  | 0.646 |
| CVLT Recognition (FP) | Mann-Whitney | 43.500 |  | 0.105 |

| **Pattern of psychometric impairment** | ***n*** | **%** |
| --- | --- | --- |
| **Seropositive** | | |
| Intact | 12 | 54.54 |
| CVLT LD (F) | 1 | 4.54 |
| VPA H | 1 | 4.54 |
| V{A E | 1 | 4.54 |
| VR2, CVLT (T), CVLT LD (F) | 1 | 4.54 |
| VR2, VPA (H) | 1 | 4.54 |
| VR1 | 1 | 4.54 |
| VR1, VR2, VPA (E), CVLT LD (F) | 1 | 4.54 |
| LM1, LM2 | 1 | 4.54 |
| LM1, LM2, VPA (E), VPA (H), | 1 | 4.54 |
| LM1, LM2, VPA (E), VPA (H), CVLT LD (F) | 1 | 4.54 |
| Total | 22 | 100 |
| **Seronegative** | | |
| Intact | 12 | 57.14 |
| VR2, CVLT LD (F) | 2 | 9.52 |
| CVLT LD (F) | 1 | 4.76 |
| VR2 | 1 | 4.76 |
| VR2, VPA(H), CVLT LD (F) | 1 | 4.76 |
| VR2, VPA(E), | 1 | 4.76 |
| LM1, LM2 | 1 | 4.76 |
| LM1, LM2, CVLT LD (F) | 1 | 4.76 |
| LM1, LM2, VR1, VR2, VPA(E), CVLT (T), CVLT LD (F) | 1 | 4.76 |
| Total | 21 | 100 |

*Note*. LM = Logical Memory; VR = Visual Reproduction; VPA = Verbal Paired Associates; CVLT = California Verbal Learning Test; F = Free Recall; LD = Long Delay; E = Easy Pair; H = Hard Pair; T = Total
